# Identifying the protective effects of miR‐874‐3p/ATF3 axis in intervertebral disc degeneration by single‐cell RNA sequencing and validation

**DOI:** 10.1111/jcmm.18492

**Published:** 2024-06-18

**Authors:** Xuke Wang, Qingfeng Wang, Guowang Li, Haiwei Xu, Bangxin Liu, Bing Yuan, Yingjie Zhou, Yongjin Li

**Affiliations:** ^1^ Department of Minimally Invasive Spine Surgery, Luoyang Orthopedic Hospital of Henan Province Orthopedic Hospital of Henan Province Luoyang Henan China; ^2^ Department of Minimally Invasive Spine Surgery Tianjin University Tianjin Hospital Tianjin China; ^3^ Department of Orthopedics The Fifth Hospital of Wuhan/The Second Affiliated Hospital of Jianghan University Wuhan China

**Keywords:** apoptosis, ATF3, ferroptosis, intervertebral disc degeneration, miR‐874‐3p, single‐cell RNA sequencing

## Abstract

Intervertebral disc degeneration (IVDD) severely affects the work and the quality of life of people. We previously demonstrated that silencing activation transcription factor 3 (ATF3) blocked the IVDD pathological process by regulating nucleus pulposus cell (NPC) ferroptosis, apoptosis, inflammation, and extracellular matrix (ECM) metabolism. Nevertheless, whether miR‐874‐3p mediated the IVDD pathological process by targeting ATF3 remains unclear. We performed single‐cell RNA sequencing (scRNA‐seq) and bioinformatics analysis to identify ATF3 as a key ferroptosis gene in IVDD. Then, Western blotting, flow cytometry, ELISA, and animal experiments were performed to validate the roles and regulatory mechanisms of miR‐874‐3p/ATF3 signalling axis in IVDD. ATF3 was highly expressed in IVDD patients and multiple cell types of IVDD rat, as revealed by scRNA‐seq and bioinformatics analysis. GO analysis unveiled the involvement of ATF3 in regulating cell apoptosis and ECM metabolism. Furthermore, we verified that miR‐874‐3p might protect against IVDD by inhibiting NPC ferroptosis, apoptosis, ECM degradation, and inflammatory response by targeting ATF3. In vivo experiments displayed the protective effect of miR‐874‐3p/ATF3 axis on IVDD. These findings propose the potential of miR‐874‐3p and ATF3 as biomarkers of IVDD and suggest that targeting the miR‐874‐3p/ATF3 axis may be a therapeutic target for IVDD.

## 1 INTRODUCTION

As a pathological condition, intervertebral disc degeneration (IVDD) is considered the pathological basis of various spinal degenerative diseases.^1^, ^2^ The IVDD‐related spinal diseases include intervertebral disc herniation, lumbar spondylolisthesis, lumbar spinal stenosis, and lumbar spondylolysis, which results in neck and shoulder pain, lower back and leg pain, and other symptoms, which severely affect the study, work and quality of life of people.^1^, ^2^, ^3^, ^4^ The incidence of IVDD diseases has recently increased in parallel with the aging population, leading to the gradual accumulation of medical costs and social burdens, which have attracted considerable attention globally.^5^, ^6^ IVDD treatment predominantly includes drug, physical and surgical therapies, which can relieve some symptoms and improve the quality of life of patients to a certain extent.^7^, ^8^, ^9^ However, it cannot block or reverse the IVDD pathological process and restore the normal structure and function of the intervertebral disc.^7^, ^8^, ^9^ Gene therapy provides promising new possibilities for the treatment of IVDD, and the effective therapeutic targets are therefore needed to be excavated.^10^ Therefore, an in‐depth study of the IVDD pathomechanism and a search for appropriate intervention targets or biomarkers to block the pathological process at the molecular level are of great significance to prevent IVDD occurrence and the development and improvement of the prognosis of IVDD diseases.^10^, ^11^

Subsequently, we analysed the pathological process and pathomechanism of IVDD. The nucleus pulposus (NP) is a vital structure of the intervertebral disc and plays a substantial role in IVDD initiation and progression.^2^, ^11^, ^12^, ^13^, ^14^ According to Risbud et al.,^13^ the IVDD pathological process can be divided into three stages, with NP cells (NPCs) being widely involved at each stage. The specific process is described as follows: NPCs first release inflammatory cytokines like TNF‐α, IL‐6 and IL‐1β in the presence of various internal and external factors, such as genetics and trauma. NPCs activate immune cells to further release pro‐inflammatory cytokines, followed by the induction of NPC death, through apoptosis and ferroptosis. Pro‐inflammatory cytokines upregulate matrix metalloproteinases (MMP) to enhance the extracellular matrix (ECM) catabolism in NPCs.^13^ Thus, changes in the NPC function, encompassing NPC death, inflammatory response, and the imbalance of ECM metabolism, are widely involved in all IVDD stages. The theoretical basis for reversing or blocking IVDD occurrence and progression through gene therapy targeting intervertebral discs includes the following three aspects: improving the adverse inflammatory microenvironment by repressing the expression of pro‐inflammatory cytokines, increasing the NPC density by elevating the NPC number in the improved microenvironment to maintain intervertebral disc stability, and promoting ECM synthesis by enhancing the expression of anabolic factors, followed by an improvement in NPC.^13^, ^14^ Thus, it is very necessary to identify the functional genes that mediate the key pathological processes of IVDD.

Non‐coding RNAs, such as microRNAs (miRNAs), have been considered crucial regulators of the gene expression over the past decades.^15^, ^16^ MiRNA has approximately 20 nucleotides, and it can inhibit protein translation by competitively binding to mRNA, without affecting the gene transcription process.^15^, ^16^ Past studies have reported that miRNAs are involved in the IVDD process by binding to the 3′‐UTR of IVDD‐related target genes, followed by mediation of the inflammatory response, NPC death, and ECM metabolism.^16^, ^17^, ^18^, ^19^ Our previous study demonstrated that miR‐874‐3p is remarkably downregulated in IVDD and negatively correlated with the severity of IVDD.^20^ Nevertheless, the biological effects and regulatory mechanisms of miR‐874‐3p in IVDD remains unclear.

In this study, we validated the biological role of miR‐874‐3p in NPCs and predicted that activation transcription factor 3 (ATF3) is its downstream target gene. ATF3 was remarkably overexpressed in IVDD, as determined through single‐cell sequencing and analysing data from relevant studies. The study also focused on the relationship between miR‐874‐3p and ATF3 by conducting molecular biology and animal experiments.

## 2 MATERIALS AND METHODS

### 2.1 Sample collection and processing

This protocol was supervised and approved by the Luoyang Orthopaedic Hospital of Henan Province and Tianjin Hospital Ethics Committee. All methods were performed according to the ARRIVE guidelines. Thirty Sprague–Dawley (SD) rats, weighing approximately 300 g, were obtained from Beijing Vital River Laboratory Animal Technology Co., Ltd. Of these, 20 rats were randomly assigned to the following four experimental groups: mimic NC, miR‐874‐3p mimic, inhibitor NC, and miR‐874‐3p inhibitor. The remaining 10 rats were enrolled in scRNA‐seq study, of which five were left untreated as control. They were anaesthetised through an intraperitoneal injection of pentobarbital sodium. A rat IVDD model was established using the needle puncture method.^20^ Subsequently, the 31G needle was used to puncture Co6–Co7 coccygeal discs and passed through the annulus fibrosus, inserted into the NP region approximately 1.5 mm along the vertical direction, rotated in the axial direction by 180°, and held for 10 s under the guidance of fluoroscopy. After 4 and 8 weeks of surgery, 2 μL of adenovirus‐encompassing mimic NC or miR‐874‐3p mimic or inhibitor NC or miR‐874‐3p inhibitor solutions were slowly injected into the experimental rats.

### 2.2 Construction of scRNA‐seq libraries and data analysis

Following the isolation of rat intervertebral disc tissues, scRNA‐seq libraries were constructed using the 10X Genomics Chromium Controller Instrument and Chromium Single Cell 3′ V3 Reagent Kits according to the suggestions. The dissociated cells were added to each channel to produce Gel Bead‐In‐Emulsions, and mRNA barcoding of single cells was performed for each sample. Subsequently, the barcoded‐cDNA was purified, amplified, fragmented and PCR amplified. The Qubit High Sensitivity DNA assay (Thermo Fisher Scientific) was conducted to quantify the final libraries. A high‐sensitivity DNA chip on a Bioanalyzer 2200 (Agilent) was used to determine the size of library distribution. All libraries were sequenced on a sequencer (Illumina, San Diego, CA) using a 150‐bp paired‐end run. After the data was dimensionally reduced through UMAP processing, Graph‐based unsupervised cell cluster results were obtained. The differentially expressed genes (DEGs) were analysed using R software Seurat package with the statistical standard was set to |log_2_ fold‐change (FC)|>2 and *p* < 0.05. R (4.2.1) software Ggplot2 package was used to generate volcano plots.

### 2.3 Immunofluorescent and TUNEL staining

Immunofluorescent and TUNEL staining need to be performed to validate the function and mechanism of miR‐874‐3p in IVDD. At 8 weeks of the puncture surgery, all rats were sacrificed by intraperitoneally injecting an overdose of pentobarbital sodium. Then, coccygeal intervertebral disc samples were obtained from the rats, fixed with 4% paraformaldehyde, decalcified in ethylene diamine tetraacetic acid, and embedded in paraffin. Subsequently, the samples were sliced into 5‐μm‐thick sections for histological assessment and staining. For immunofluorescence analysis, the sections were incubated with the primary antibodies anti‐ATF3 (Abcam, ab254268) and anti‐GPX4 (Abcam, ab125066) overnight at 4°C, followed by incubation with a secondary antibody for 60 min at room temperature. To measure NPC apoptosis, TUNEL staining was performed using an apoptosis detection kit (keyGEN, Jiangsu, China) according to the manufacturer protocol. The nuclei were stained with 4,6‐diamidino‐2‐ phenylindole (DAPI). All images were captured using a fluorescence microscope (Leica).

### 2.4 Obtaining the ferroptosis‐related DEGs

We obtained 254 ferroptosis‐related genes from the FerrDb V2 database (http://www.zhounan.org/ferrdb/), including ferroptosis inducers, inhibitors, markers and unclassified genes.^21^ The DEGs in fibrochondrocyte progenitors (FCPs) and homeostatic chondrocytes (HomCs) were obtained.^22^ To explore the key ferroptosis genes in IVDD, Venn analysis was performed to identify ferroptosis‐related DEGs through the intersection of FCPs, HomCs and ferroptosis genes.

### 2.5 Predicting the target gene of miR‐874‐3p

We obtained ECM and apoptosis genes from the literatures.^19^, ^23^ The target gene of miR‐874‐3p was predicted by mirDIP (https://ophid.utoronto.ca/mirDIP/index.jsp) database (Score class: Very High Top 1%).^24^ To identify the key functional target gene of miR‐874‐3p in IVDD, Venn analysis was performed to select the overlapping gene through the intersection of ECM genes, apoptosis genes, ferroptosis genes and mirDIP database.

### 2.6 Analysis of protein–protein interaction network and the identification of hub gene

We constructed a protein–protein interaction (PPI) network to further identify the key hub ferroptosis gene. The STRING website summarizes extensive data on the interaction between proteins. The difference in the strength of the connection between proteins can be obtained according to the strength of the evidence.^25^ This network data node and target data to the local were downloaded from the STRING website and imported into Cytoscape software. Cytoscape is an open software platform used to visualize molecular interaction networks. The connection strength of gene nodes can be scored through the analysis conducted by the cytohubba plug‐in in the Cytoscape software. Finally, the gene with the highest score was considered the hub gene.^26^

### 2.7 Gene Ontology functional and Kyoto Encyclopedia of Genes and Genomes pathway enrichment analysis

Gene functional enrichment analysis is a highly favoured and used bioinformatics analysis tool by researchers, mainly including Gene Ontology (GO) functional annotation and Kyoto Encyclopedia of Genes (KEGG) pathway enrichment analysis. To predict the potential functions of the ferroptosis‐related DEGs, we conducted gene functional enrichment analysis. GO is divided into three categories, namely biological process (BP), cell components (CC) and molecular function (MF). The KEGG analysis was performed to determine the main signalling pathways. These categories are used to elucidate the involvement of key DEGs in mediating signalling pathways or biological processes, thereby revealing the potential role and possible mechanisms of DEGs in IVDD.

### 2.8 NPC culture

As described previously,^20^ human primary NPC isolated from the human normal degenerative disc were obtained from ScienCell Research Laboratories (Sciencell, USA). The NPC were cultured in Nucleus Pulposus Cell Medium (Sciencell). The medium was placed in a humid environment at 37°C and 5% CO_2_. The NPC with good growth conditions were used for all subsequent experiments.

### 2.9 Vector construction and NPC transfection

The ATF3 small‐interfering RNA (siRNA) and miR‐874‐3p mimic/inhibitor (both Beyotime, China) were transfected into NPC using Lipofectamine 3000 (Beyotime, Shanghai, China) according to the manufacturer's suggestion. At 48 h after transfection, NPC were used for performing the subsequent experiments.

### 2.10 Western blotting experiment

We performed Western blotting experiment to validate the regulatory mechanism. Briefly, we extracted proteins from NPC by using RIPA lysis buffer with phenylmethanesulfonylfluoride. Subsequently, SDS‐PAGE was performed to isolate the proteins, which were then transferred onto polyvinylidene difluoride (PVDF) membranes. The membranes were covered for 1 h, incubated overnight with primary antibodies at 4°C, and washed with PBS, for 10 min each. Then, the PVDF membranes were incubated with a secondary antibody for 1 h at room temperature. Next, the membranes were placed into a chemiluminescent substrate for development. The luminous signals were detected using the chemiluminescence system (Bio‐Rad, CA, USA). The primary antibodies used were anti‐ATF3 (Abcam, ab254268), anti‐GPX4 (Abcam, ab125066), anti‐Aggrecan (Abcam, ab3778), anti‐COL2A1 (Abcam, ab188570), anti‐SLC7A11 (Abcam, ab175186), anti‐Caspase‐3 (Abcam, ab32351), anti‐MMP2 (Abcam, ab92536), anti‐MMP3 (Abcam, ab52915), and anti‐GAPDH (Abcam, ab8245).

### 2.11 Flow cytometry

NPC apoptosis was measured using the Annexin V‐FITC apoptosis detection kit (keyGEN, Jiangsu, China) under different processing conditions. Then, NPC in different apoptosis stages, including the normal surviving NPCs, early apoptotic NPCs, and late apoptotic and necrotic NPCs, were separated by staining them with Annexin V‐FITC and 7‐AAD. Subsequently, Flowjo VX10 software was used to analyse the data. The early apoptotic NPCs (Q3) and late apoptotic and necrotic NPCs (Q2) indicated NPC apoptosis.

### 2.12 Enzyme‐linked immunosorbent assay and reactive oxygen species detection

To measure the TNF‐α, IL‐6 and IL‐1β levels, respectively, under different conditions in NPC, we obtained TNF‐α, IL‐6 and IL‐1β ELISA kits (Elabscience). More specifically, TNF‐α, IL‐6, and IL‐1β antibodies were added to the enzyme‐linked immunosorbent assay (ELISA) assay well, and standards and samples were added to the microplates. Each standard and sample were measured through ELISA at 450 nm. The TNF‐α, IL‐6 and IL‐1β levels were inferred based on the absorbance value. The reactive oxygen species (ROS) level in human NPCs was detected using the ROS assay kit (Elabscience) following the manufacturer's suggestions.

### 2.13 Dual‐luciferase reporter gene experiment

Based on the MirDIP database,^24^ we found the binding sites between miR‐874‐3p and ATF3 3′‐UTR. Dual‐luciferase reporter vectors psiCHECK2‐Firefly luciferase‐Renilla luciferase containing ATF3 wild type sequence or mutant type sequence were purchased from Guangzhou Geneseed Biotech Co (Guangzhou, China). Then, 1 μg wild type or mutant type vectors and 100 μL miR‐874‐3p inhibitor or inhibitor NC were co‐transfected to HEK‐293 T cells by lipofectamine 3000 (Beyotime, China). We used the Dual‐luciferase Assay Kit (Beyotime, China) to detect the luciferase activity of ATF3 wild type and mutant type. The value of luciferase activity was reckoned according to the obtained ratio of the relative light unit value detected by Renilla luciferase is divided by the relative light unit value detected by firefly luciferase.

### 2.14 Statistical analysis

The data were analysed and figures were drawn using GraphPad Prism software 8 version. All experiments were conducted at least thrice. The statistical significance between the groups was compared using the unpaired Student's *t‐*test, while differences among more than two groups were assessed using one‐way ANOVA, followed by Turkey's multiple comparison test. The results were presented as the mean ± standard deviation (SD). *p* < 0.05 was determined to indicate statistical significance. *p < 0.05; **p < 0.01 and ***p < 0.001.

## 3 RESULTS

### 3.1 ATF3 was a target gene of miR‐874‐3p

**FIGURE 1:**
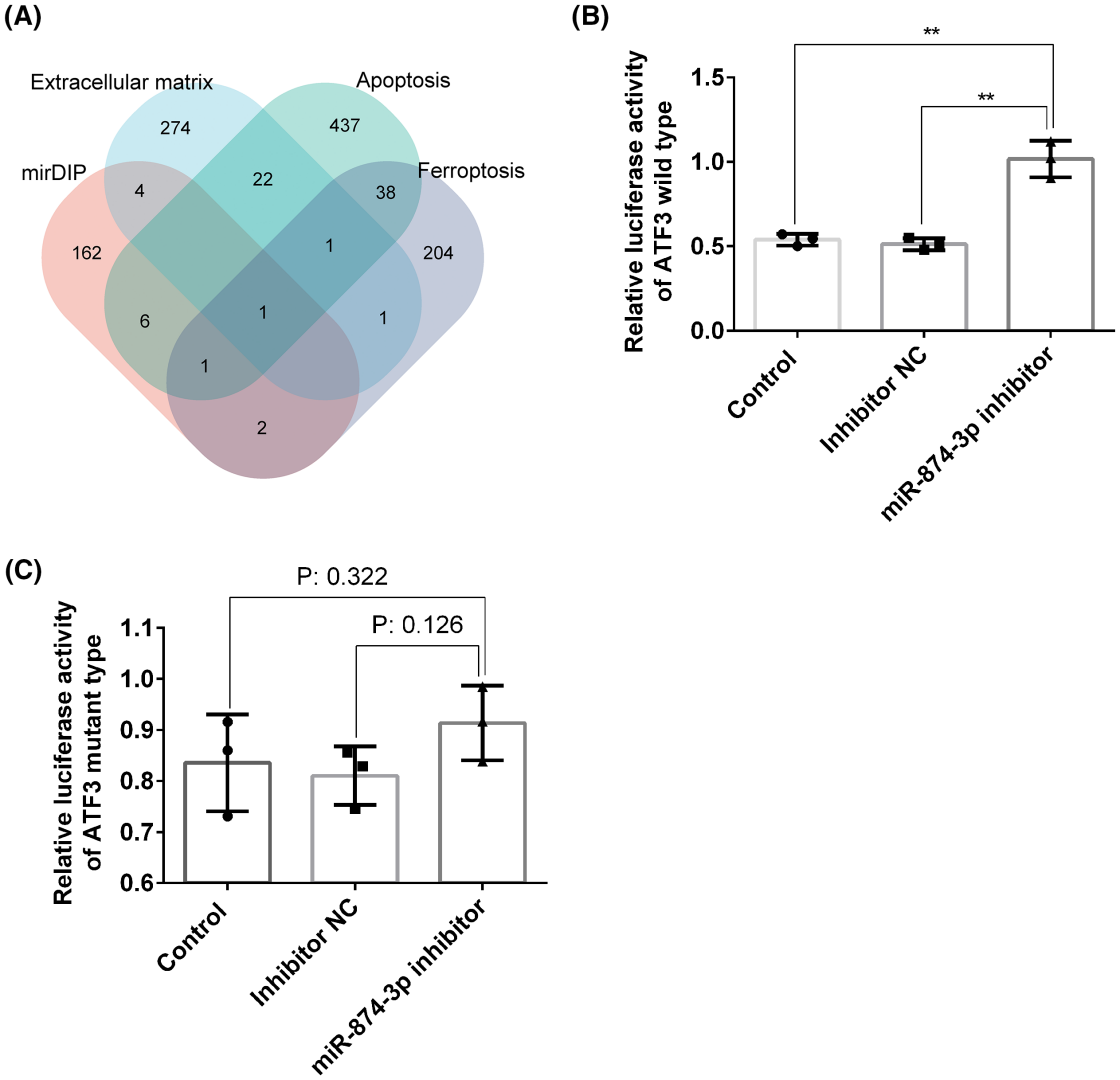
ATF3 had been proven to be the target gene of miR‐874‐3p. (A) Venn plot predicted that ATF3 is the target gene of miR‐874‐3p. (B, C) Dual‐luciferase reporter gene experiment proved that miR‐874‐3p directly binds to ATF3. **P ＜ 0.01.

MirDIP is a well‐known database that collects miRNA‐gene interactions from multiple databases, such as miRNATIP, TargetScan, miranda and miRDB.^24^ We used mirDIP database to predict the target genes of miR‐874‐3p. We identified a key functional target gene of miR‐874‐3p, ATF3, by aggregating ferroptosis genes, ECM genes, apoptosis genes and target genes obtained from mirDIP database (Figure 1A). As is well known, miRNAs generally interact with target genes by binding to their 3′‐UTR. To test this, we conducted dual‐luciferase reporter gene experiment in HEK‐293 T cells. The results proved that miR‐874‐3p inhibitor significantly increased the luciferase activity of ATF3 wild type (Figure 1B), whereas it had little effect on the luciferase activity of ATF3 mutant type (Figure 1C), suggesting that miR‐874‐3p directly binds to the 3′‐UTR of ATF3. These experimental data confirmed that ATF3 was the target gene of miR‐874‐3p.

### 3.2 ATF3 was remarkably upregulated in IVDD patients

**FIGURE 2:**
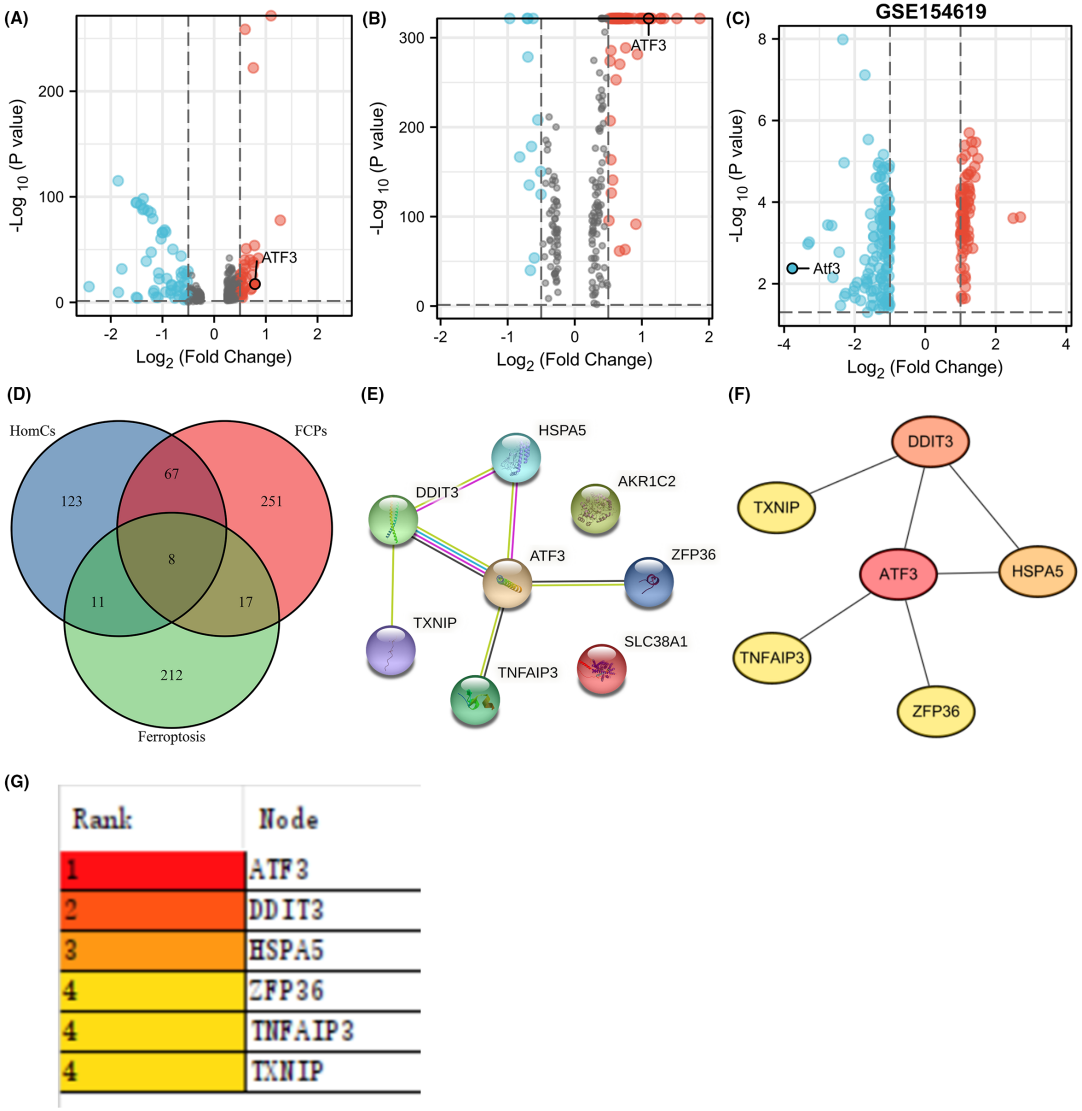
ATF3 was a key ferroptosis gene and highly expressed in IVDD patients. (A) The expression of ATF3 in human homeostatic chondrocytes (HomCs). (B) The expression of ATF3 in human fibrochondrocytes progenitors (FCPs). (C) Volcano plot showing that *Atf3* was the most downregulated gene in GSE154619. (D) Venn diagram analysis of overlapping genes in FCPs, HomCs, and ferroptosis genes. (E–G) PPI analysis of 8 overlapping genes.

Based on Pfirrmann's grading, Zhang et al.^22^ found that chondrocytes accounted for 99.33% by performing scRNA‐seq on 1 normal, 3 moderate, and 2 severe degenerative human NP tissues. Seven chondrocyte subtypes were observed in the NP according to differential gene expression, namely FCPs, HomCs, cartilage progenitor cells, and four novel populations (C1–C4). Intriguingly, ATF3 was present in six of these chondrocyte subtypes and was particularly highly expressed in degenerative FCPs and HomCs (Figure 2A,B). Furthermore, Novais et al.^27^ demonstrated that the dasatinib and quercetin drug combination could inhibit age‐dependent IVDD development in mice and conducted microarray analysis. Data from the GSE154619 dataset were analysed, and Atf3 downregulation was found to be most pronounced in the intervertebral discs of mice treated with the aforementioned combination (Figure 2C). This finding suggests that dasatinib and quercetin may prevent IVDD development by inhibiting the Atf3 expression. Subsequently, 8 ferroptosis‐related DEGs were found through the intersection of ferroptosis genes, FCPs, and HomCs (Figure 2D). The STRING website summarizes a large amount of data on the interaction between proteins. The difference in the strength of the connection between proteins can be obtained according to the strength of the evidence.^25^ These 8 DEGs were input into the STRING website to obtain a network diagram of PPIs (Figure 2E). The PPI network data, including ATF3, TXNIP, TNFAIP3, SLC38A1, ZFP36, AKR1C1, HSPA5 and DDIT3, were imported into Cytoscape software. According to the cytohuba plugin of Cytoscape, ATF3 was identified as the most critical gene in the protein interaction network (Figure 2F,G). Bioinformatics analysis revealed that ATF3 was a key ferroptosis and apoptosis gene and remarkably upregulated in IVDD patients.

### 3.3 Atf3 was highly expressed in multiple cell types of rat‐degenerated intervertebral discs

**FIGURE 3:**
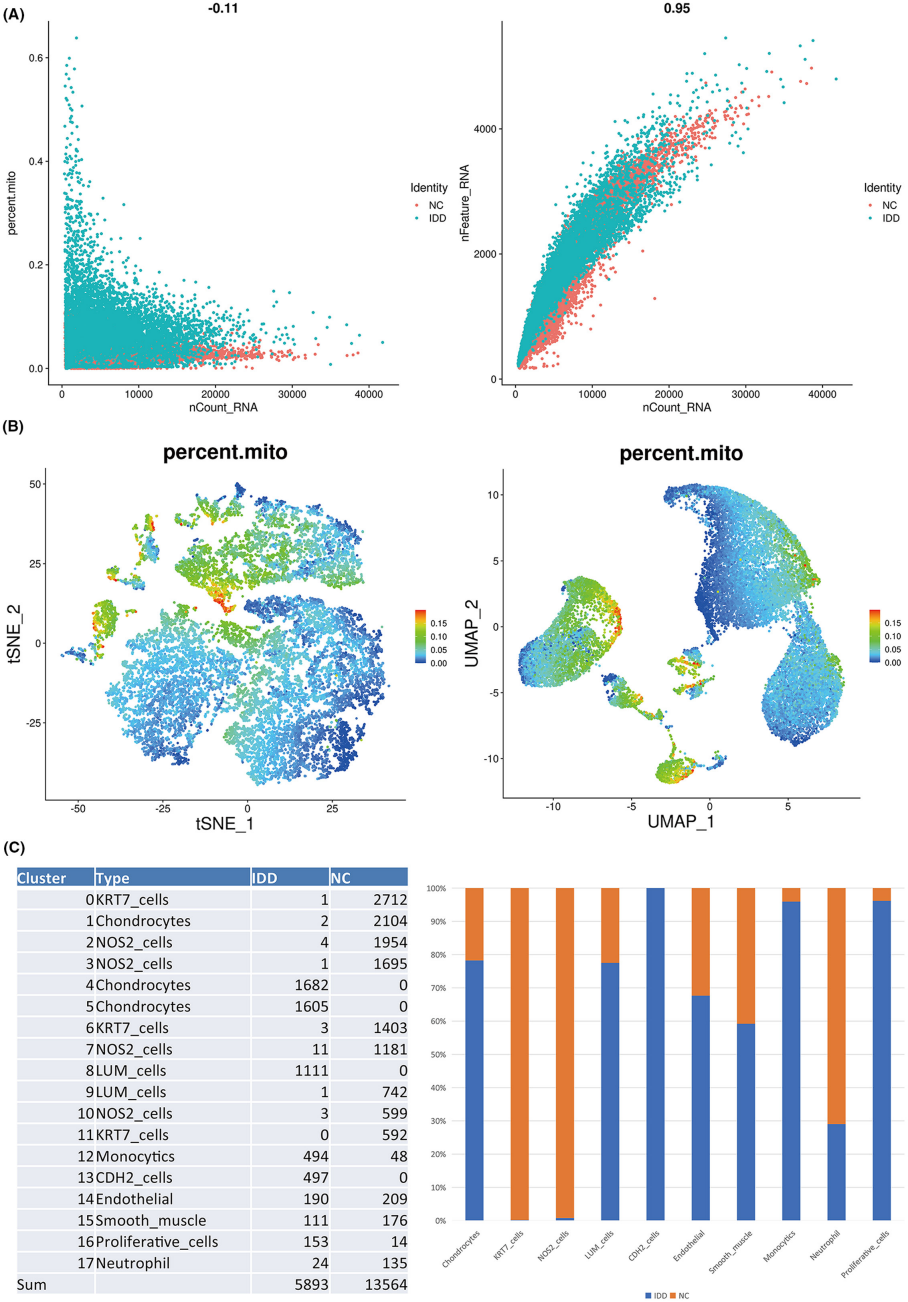
Single‐cell RNA sequencing identifies relevant cell populations in normal and degenerated rat intervertebral discs. (A) Correlational analysis of the mitochondrial ratio and genes number per cell‐UMI number in single‐cell sequencing. (B) The proportion of mitochondria in each cell in the T‐distributed Stochastic Neighbour Embedding (t‐SNE) diagram and Uniform Manifold Approximation and Projection (UMAP) diagram. (C) Specific numbers and proportions of different cell types in normal and degenerated intervertebral discs in rats.

**FIGURE 4:**
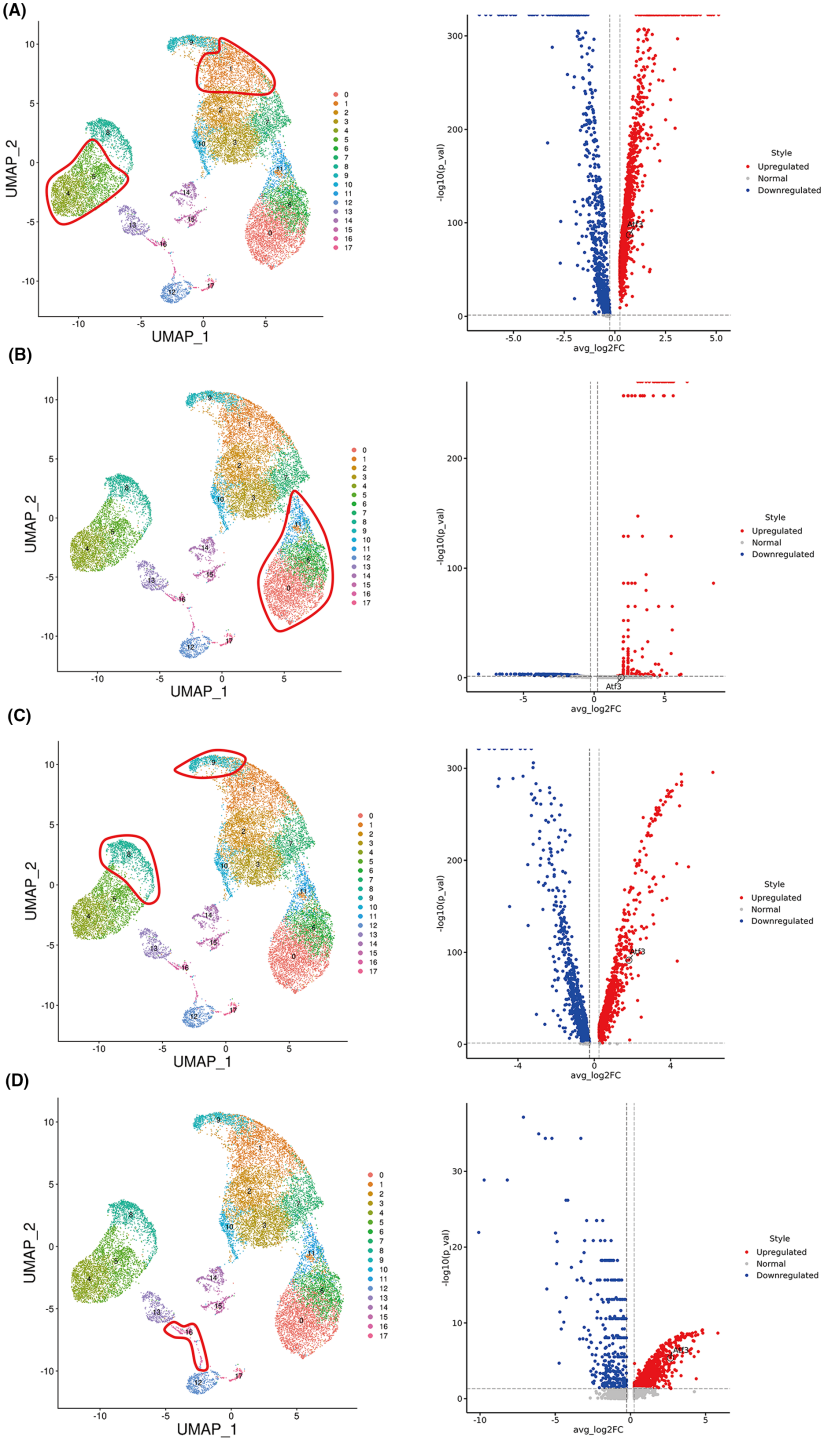
Single‐cell RNA sequencing identified Atf3, which was highly expressed in various cell types of rat‐degenerated intervertebral discs. (A) Cell populations 1, 4, and 5 are chondrocytes, Atf3 was remarkably overexpressed in NP chondrocytes of degenerated intervertebral discs when compared with that in normal rat intervertebral discs. (B) Cell populations 0, 6, and 11 are KRT7+ cells, and Atf3 was remarkably overexpressed in the NP‐inflammatory response of degenerated intervertebral discs. (C) Cell populations 8 and 9 are LUM+ cells, and Atf3 has remarkably overexpressed in the extracellular matrix (ECM) NP cells of degenerated intervertebral discs. (D) Cell population 16 is proliferating cells, and Atf3 was remarkably overexpressed in the proliferating cells of degenerated intervertebral discs.

NPCs are heterogeneous, and multiple types of NPCs are formed. However, their type composition and fate during IVDD remain poorly understood.^28^ To identify the NPC subtypes, we performed single‐cell RNA sequencing on the rat intervertebral discs. As shown in Figure 3A,B, the results of data quality control display that the cell activity and quality were good and the gene number was appropriate, which was therefore used for further analysis. Compared with the normal intervertebral discs, 18 clusters were found in the degenerated intervertebral discs (Figure 3C). Among them, the following 10 cell types were identified: chondrocytes, KRT7_cells, NOS2_cells, LUM_cells, CDH2_cells, monocytics, endothelial cells, smooth_muscle cells, proliferative_cells and neutrophils (Figure 3C). Figure 3C presents the proportion of these 10 cell types in IVDD. Of them, chondrocytes accounted for the highest proportion, which is consistent with previous study findings.^22^, ^29^, ^30^ Atf3 was remarkably overexpressed to varying degrees in chondrocyte clusters (1, 4 and 5) (Figure 4A), ECM‐secreting LUM+ cell clusters (8 and 9) (Figure 4B), inflammatory response‐associated KRT7+ cell clusters (0, 6 and 11) (Figure 4C), and the proliferation‐associated Ki67+ cell cluster (16) (Figure 4D) of the degenerated intervertebral discs compared with the normal rat intervertebral discs.

### 3.4 Predicting the biological functions of ATF3 by GO functional and KEGG pathway enrichment analysis

**FIGURE 5:**
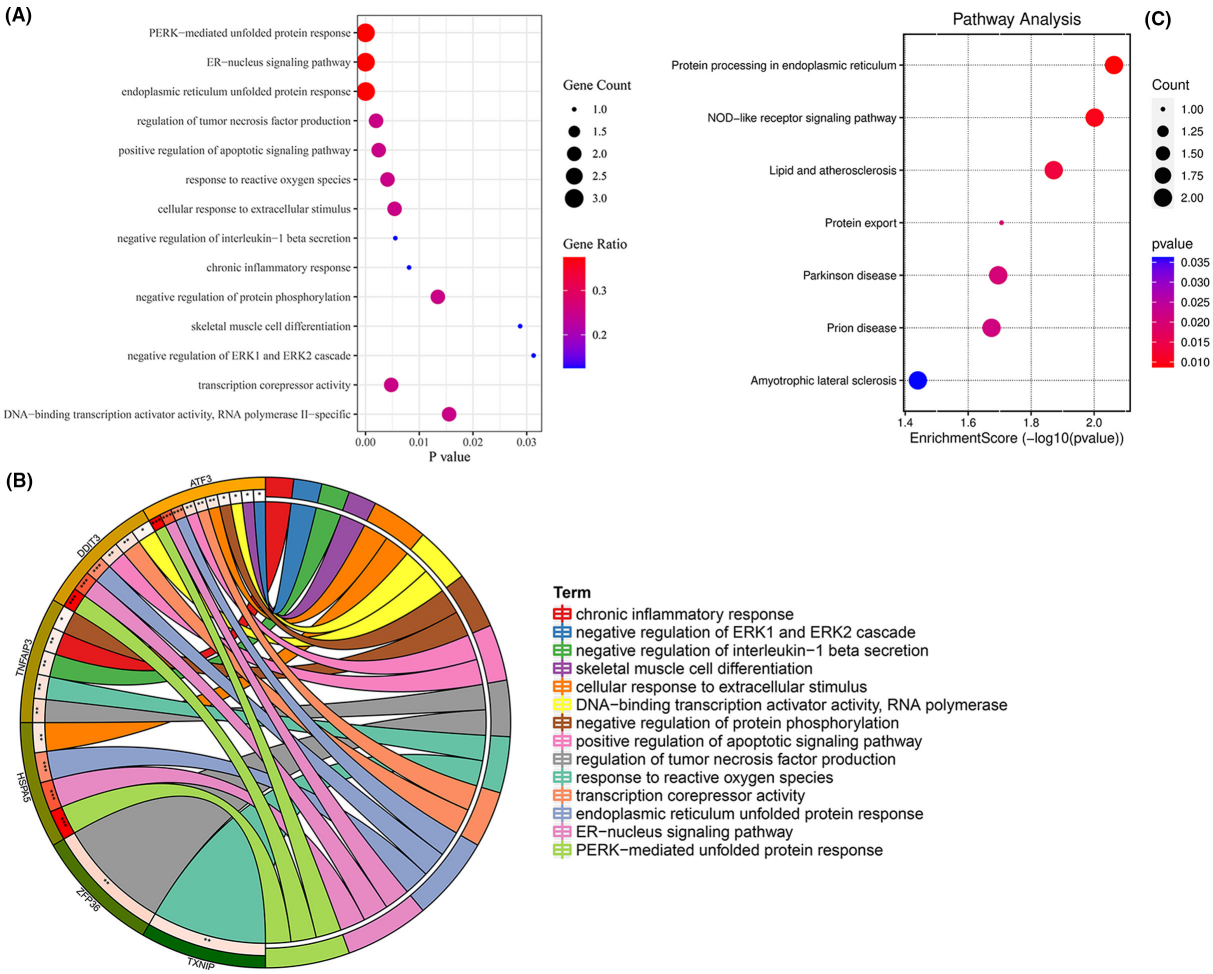
GO and KEGG enrichment analyses. (A, B) The results of GO functional enrichment analysis are indicated by bubble diagrams and GO chord diagram. (C) KEGG pathway enrichment analysis of 8 ferroptosis genes.

To elucidate the potential biological functions of the abovementioned 8 ferroptosis genes, we conducted GO function and KEGG pathway enrichment analysis. The 15 GO related to the study were enriched in the ‘regulation of tumor necrosis factor production, negative regulation of interleukin‐1 beta secretion, response to ROS, chronic inflammatory response, positive regulation of apoptotic signaling pathway, cellular response to extracellular stimulus, PERK‐mediated unfolded protein response, ER‐nucleus signaling pathway, endoplasmic reticulum unfolded protein response, negative regulation of protein phosphorylation, skeletal muscle cell differentiation, negative regulation of ERK1 and ERK2 cascade, transcription corepressor activity, DNA‐binding transcription activator activity, and RNA polymerase II‐specific’ (Figure 5A,B). GO chord diagram further unveiled that ATF3 might be involved in the regulation of cell apoptosis, cell differentiation and ECM metabolism (Figure 5C). The KEGG analysis discovered that these genes were mainly involved in protein processing in the ER, NOD‐like receptor signalling pathway, lipid and atherosclerosis, protein export, Parkinson's disease, prion disease, amyotrophic lateral sclerosis (Figure 5C). These findings suggested that ATF3 may regulate the progression of IVDD by mediating a series of pathological processes, albeit the regulatory mechanism remain unclear.

### 3.5 MiR‐874‐3p repressed NPC apoptosis, ferroptosis and ECM degradation by targeting ATF3

**FIGURE 6:**
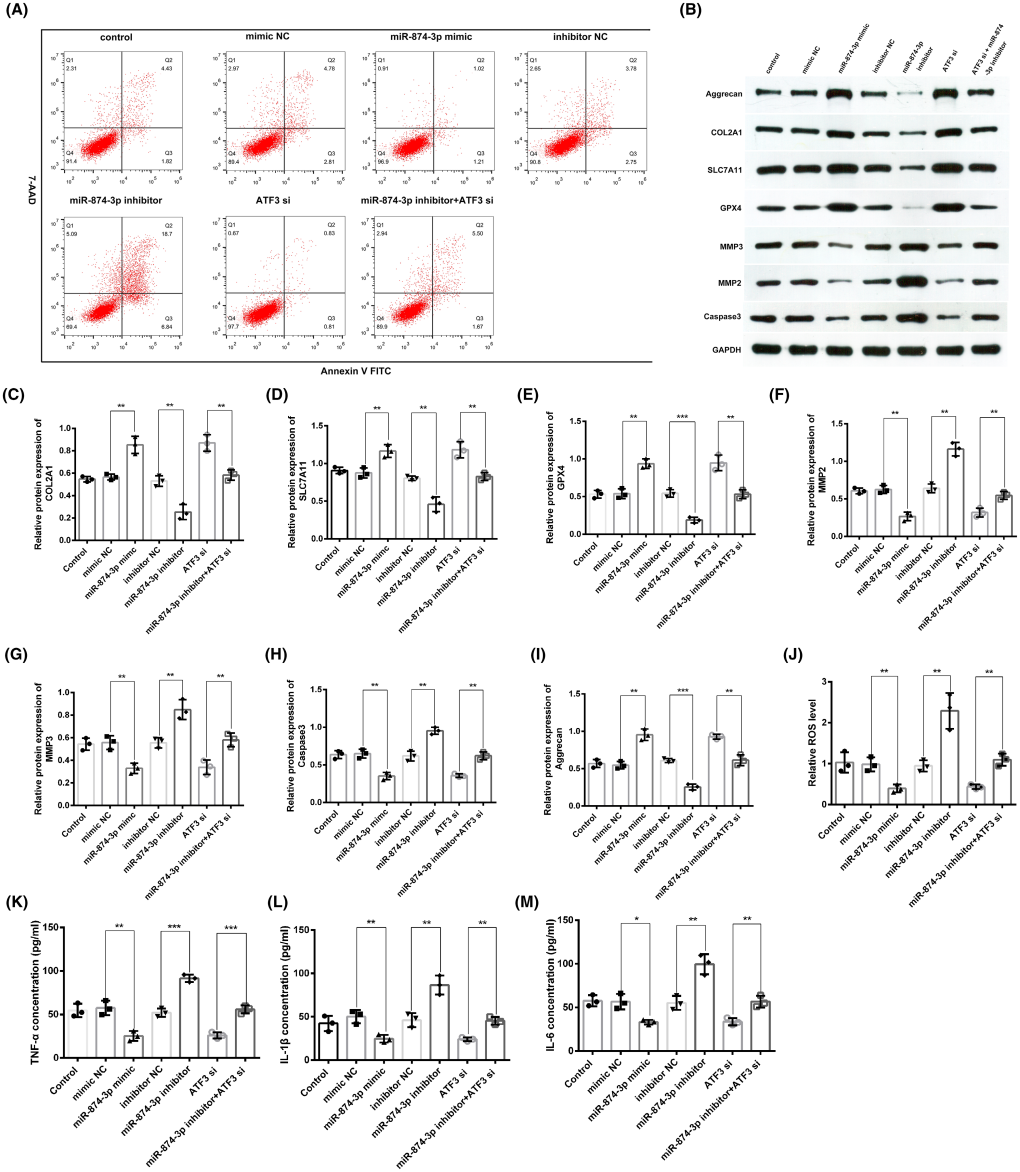
MiR‐874‐3p repressed NPCs apoptosis, ferroptosis, inflammatory response, and ECM degradation by targeting ATF3. (A) Flow cytometry detected the NPCs apoptosis under different conditions. (B–I) The protein expressions of SLC7A11, GPX4, Aggrecan, COL2A1, caspase3, MMP2, and MMP3 under different conditions as detected by Western blotting in NPCs. (J) The relative ROS levels under different conditions. (K–M) ELISA was performed to detect the concentration of TNF‐α, IL‐1β, and IL‐16 in NPCs under different conditions. *p < 0.05, **p < 0.01, ***p < 0.001.

We intend to study the biological effects of miR‐874‐3p in IVDD. To establish miR‐874‐3p‐overexpressing or miR‐874‐3p/ATF3‐knockdown NPCs, the miR‐874‐3p mimic or miR‐874‐3p inhibitor or ATF3 siRNA was transfected into NPCs, respectively. Gain‐of‐function and loss‐of‐function experiments unveiled that miR‐874‐3p overexpression remarkably inhibited NPC apoptosis, whereas miR‐874‐3p knockdown remarkably enhanced NPC apoptosis. ATF3 silencing inhibited NPC apoptosis and reversed the effect of the miR‐874‐3p inhibitor on NPC apoptosis (Figure 6A). Moreover, the levels of the apoptosis marker caspase3 were remarkably reduced in the miR‐874‐3p‐overexpressing and ATF3‐knockdown NPCs, whereas remarkably increased in miR‐874‐3p‐knockdown NPCs (Figure 6B,H). The MMP2 and MMP3 protein expressions remarkably reduced and SLC7A11, GPX4, Aggrecan and COL2A1 protein expressions remarkably increased in the miR‐874‐3p‐overexpressing and ATF3‐knockdown NPCs, while miR‐874‐3p knockdown led to the opposite NPC phenotypes (Figure 6B–I). In the NPCs co‐transfected with the miR‐874‐3p inhibitor and ATF3 siRNA, the altered expression of these proteins was reversed to the normal levels, which is consistent with our expected result (Figure 6B–I). The rescue experiments revealed that the caspase3 level‐promoting effect of the miR‐874‐3p inhibitor was counteracted by the caspase3 level‐suppressing effect of ATF3 (Figure 6B,H). Furthermore, the miR‐874‐3p mimics remarkably repressed the ROS levels, whereas the miR‐874‐3p inhibitor remarkably promoted the ROS levels in NPCs (Figure 6J). Notably, the ROS level‐promoting effect of the miR‐874‐3p inhibitor was counteracted by the ROS level‐suppressing effect of ATF3 siRNA (Figure 6J). Taken together, these data suggested that miR‐874‐3p might repress NPC apoptosis, ferroptosis, and ECM degradation by targeting ATF3.

### 3.6 MiR‐874‐3p repressed the inflammatory response by targeting ATF3

The inflammatory response plays a crucial role in mediating the IVDD pathological process. Thus, ELISA was performed to measure the levels of TNF‐α, IL‐6 and IL‐1β—the most studied pro‐inflammatory cytokines^3^, ^13^—after transfecting the miR‐874‐3p mimic or miR‐874‐3p inhibitor or NC or ATF3 siRNA or miR‐874‐3p inhibitor+ATF3 siRNA into NPCs. The TNF‐α, IL‐6 and IL‐1β expressions were remarkably increased in the NPCs with miR‐874‐3p knockdown, whereas miR‐874‐3p overexpression or ATF3 knockdown remarkably reduced their levels (Figure 6K–M). In addition, the rescue experiments demonstrated that their expressions exhibited no significant alteration in NPCs co‐transfected with the miR‐874‐3p inhibitor and ATF3 siRNA. These results thus suggested that miR‐874‐3p represses the inflammatory response by targeting ATF3. Thus, these findings confirmed that miR‐874‐3p possibly protects against IVDD by inhibiting NPC ferroptosis, apoptosis, ECM degradation and inflammatory response by targeting ATF3 and its downstream genes.

### 3.7 MiR‐874‐3p might repress IVDD progression by targeting ATF3 in a rat model

**FIGURE 7:**
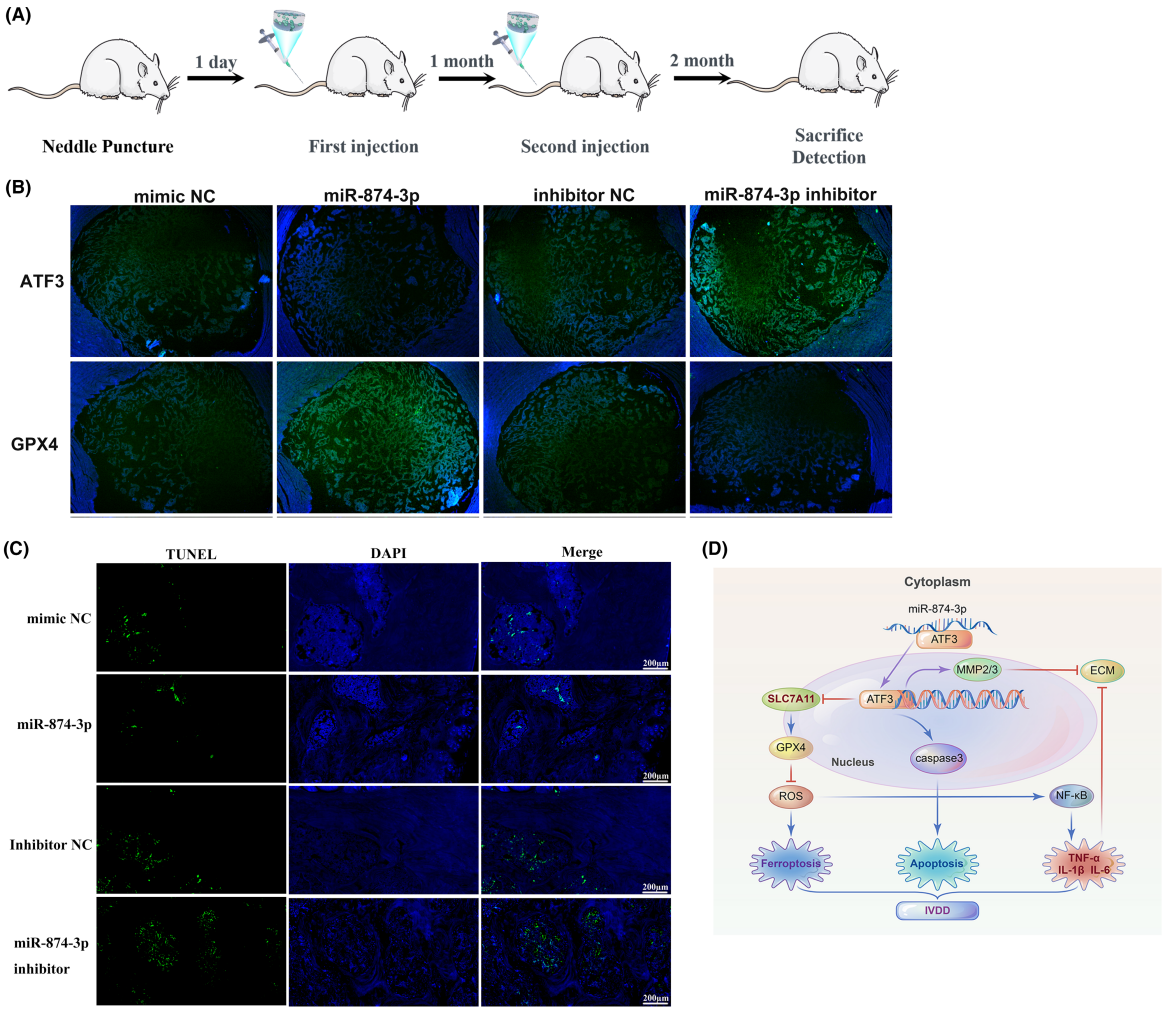
MiR‐874‐3p may repress IVDD in a rat model. (A) Animal experiment flow‐process diagram. (B) Immunofluorescence staining measured the expression of ATF3 and GPX4 in rats under different conditions. (C) TUNEL staining demonstrated that the overexpression of miR‐874‐3p was inhibited, whereas the knockdown of miR‐874‐3p enhanced NPCs apoptosis. (D) Schematic diagram of the potential mechanism via the miR‐874‐3p/ATF3 signalling axis in the occurrence and development of IVDD. MiR‐874‐3p may protect against IVDD by inhibiting NPCs ferroptosis, apoptosis, ECM degradation, and inflammatory response via targeting ATF3 and its downstream genes.

To confirm the underlying roles and molecular mechanisms of miR‐874‐3p in IVDD, the IVDD model was constructed in wild‐type rats through needle puncture of the rat caudal vertebrae, followed by a local injection of the miR‐874‐3p mimic/inhibitor at 1 day and 1 month after surgery (Figure 7A). The ATF3 expression was remarkably reduced, whereas the GPX4 expression was remarkably increased in the miR‐874‐3p‐treated rats. Conversely, the miR‐874‐3p inhibitor‐treated rats exhibited a higher ATF3 expression and a lower GPX4 expression (Figure 7B). Furthermore, TUNEL staining further demonstrated that miR‐874‐3p overexpression inhibited NPC apoptosis in the miR‐874‐3p‐treated rats, whereas miR‐874‐3p knockdown enhanced NPC apoptosis in the miR‐874‐3p inhibitor‐treated rats (Figure 7C), suggesting that the miR‐874‐3p overexpression has a protective effect against IVDD. These findings thus implied that miR‐874‐3p might repress IVDD progression by targeting ATF3 in a rat model. Figure 7D presents a schematic diagram of the potential mechanism of the miR‐874‐3p/ATF3 axis involved in IVDD occurrence and development.

## 4 DISCUSSION

The aetiology of IVDD is multifactorial, including genetics, smoking and aging.^13^, ^31^, ^32^ Growing evidence suggests genetics to be a major causative factor for IVDD.^31^, ^32^ The pathogenic mechanism underlying IVDD has been deeply explored to reveal the genetic cause of IVDD. Epigenetics is a branch of genetics involving the study of heritable changes in the gene expression without any change in the gene nucleotide sequence, and its research scope includes the study of miRNA and mRNA.^33^ Our group previously demonstrated that miRNA and mRNA evidently influence the IVDD pathological process.^17^, ^19^, ^34^, ^35^ However, to date, no key miRNAs have been found in IVDD that can regulate ferroptosis and apoptosis, as well as mediate ECM synthesis and inhibit the secretion of pro‐inflammatory factors. In our study, we found that ATF3 is a direct target of miR‐874‐3p, and miR‐874‐3p/ATF3 signalling is dysregulated during IVDD. Thus, targeting miR‐874‐3p/ATF3 signalling axis might be potential therapeutic targets for IVDD.

NP has been known to exert a central role in IVDD pathomechanism. In fact, NP is a heterogeneous tissue with multiple cell types.^36^ However, their type composition and development route in the IVDD process remain poorly understood. Thus, additional studies are warranted to comprehensively understand the cellular heterogeneity and molecular mechanisms related to IVDD. ScRNA‐seq is considered an invaluable tool for exploring cellular heterogeneity and fate.^22^, ^29^, ^30^, ^36^, ^37^ In this study, we obtained the rat tail disc tissues for scRNA‐seq. Atf3 was remarkably upregulated in various cell types of the rat degenerated intervertebral discs, including chondrocyte clusters, ECM‐secreting LUM+ cell clusters, inflammatory response‐associated KRT7+ cell clusters, and proliferation‐associated Ki67+ cell clusters. Researches have discovered that chondrocytes account for the highest proportion in intervertebral disc tissues by scRNA‐seq, with three types: regulatory chondrocytes, HomCs, and effector chondrocytes.^22^, ^29^ Furthermore, ATF3 was remarkably upregulated in human FCPs and HomCs. These data indicated that ATF3 is indeed a key gene in the IVDD process.

Dixon et al.^38^ were the first to report an iron‐dependent cell death process, ferroptosis, in the *Cell* Journal. Ferroptosis is characterized by cellular lipid peroxidation and increased ROS production.^38^, ^39^ ROS are crucial intermediators in the IVDD‐related signalling pathway. They possess a crucial role in IVDD by regulating ECM metabolism, inflammatory response and death of NPCs.^40^ Ferroptosis plays a crucial role in IVDD and contributes to the pathomechanism of the IVDD.^20^, ^22^, ^41^, ^42^, ^43^, ^44^ Through scRNA‐seq, Zhang et al.^22^ noted that multiple ferroptosis‐related genes are remarkably upregulated in IVDD patients. They demonstrated that these ferroptosis indicators were remarkably increased in the rat IVDD model, which indicated that ferroptosis occurs in the IVDD process. SLC7A11 and GPX4 are typical ferroptosis markers that can suppress ferroptosis.^45^, ^46^, ^47^ Yang et al.^41^ found that the expressions of the ferroptosis markers GPX4 and FTH were remarkably reduced, whereas those of PTGS2 and ACSL4 were remarkably elevated in IVDD. NCOA4 silencing repressed NPC ferroptosis in an autophagy‐dependent manner.^41^ Melatonin was reported to reduce lipopolysaccharide‐induced NPC ferroptosis in a dose‐dependent manner, manifested as elevated levels of GPX4 and SLC7A11.^42^ According to Wang et al.,^43^ iron overload aggravates IVDD progression by promoting ferroptosis. FPN reduces intracellular iron accumulation and protects NPCs from ferroptosis.^44^ ATF3 inhibition could protect against IVDD by repressing SLC7A11‐mediated ferroptosis.^20^ Thus, ferroptosis has been implicated in IVDD. Nevertheless, it is not yet known whether miR‐874‐3p affects IVDD by mediateing ferroptosis. This study first demonstrated that miR‐874‐3p could repress ferroptosis and ROS production by targeting ATF3 and it target genes GPX4 and SLC7A11 through in vitro and in vivo experiments.

MMP2 and MMP3 are crucial MMP family members and can promote ECM degradation.^11^, ^12^, ^13^ Caspase3 is a marker and an executor of apoptosis.^48^ Our group revealed that the circ_0040039‐miR‐874‐3p‐ESR1 signalling axis might be implicated in IVDD. The group also verified that circ_0040039 promotes the ESR1 expression by inhibiting the miR‐874‐3p expression in NPCs. This finding indicated that miR‐874‐3p might offer protection in IVDD, but the regulatory mechanism remains unclear.^49^ Furthermore, Yang et al. demonstrated that miR‐874‐3p represses NPC apoptosis by decreasing caspase3 and ECM degradation by reducing MMP2 and MMP3.^50^ In this study, we demonstrated that miR‐874‐3p protect against IVDD by inhibiting NPC ferroptosis, apoptosis, ECM degradation and inflammatory response through the regulation of SLC7A11, GPX4, caspase3, MMP2, MMP3, TNF‐α, IL‐6 and IL‐1β expression in NPC. Moreover, animal experiments also supported the above conclusion.

However, our study has some limitations. First, we analysed the scRNA‐seq data from human NP tissues through a literature search. We performed scRNA‐seq using intervertebral disc tissues from rat tails, but not from humans. Fluorescence‐activated cell sorting strategy need to be performed to isolate the key chondrocyte cluster from nucleus pulposus tissues. Then, we can validate the roles and regulatory mechanisms of miR‐874‐3p/ATF3 signalling axis in this chondrocyte cluster. Second, we only collected one normal and one degenerative intervertebral disc tissue for sequencing. This small sample size may affect the accuracy of the results. More samples are needed for sequencing and validation in the future. Finally, animal experiments such as imaging and histological evaluation methods need to be conducted to demonstrate the regulatory effects of the miR‐874‐3p/ATF3 axis in IVDD.

## 5 CONCLUSION

Through scRNA‐seq, we here found that ATF3 is a direct target of miR‐874‐3p, and Atf3 was highly expressed in chondrocyte clusters, ECM‐secreting LUM+ cell clusters, inflammatory response‐associated KRT7+ cell clusters, and proliferation‐associated Ki67+ cell clusters of degenerated intervertebral discs of rats. Moreover, ATF3 was remarkably upregulated in human FCPs and HomCs. Furthermore, miR‐874‐3p might protect against IVDD through the inhibition of NPC ferroptosis, apoptosis, ECM degradation, and inflammatory response by targeting ATF3 and its downstream genes. Moreover, experiments in rats also revealed that miR‐874‐3p might repress IVDD progression by targeting ATF3. These findings unveiled that the miR‐874‐3p/ATF3 axis exerted a substantial role in IVDD, highlighting that miR‐874‐3p and ATF3 might serve as potential therapeutic targets for IVDD.

## AUTHOR CONTRIBUTIONS

**Xuke Wang:** Writing – original draft (lead). **Qingfeng Wang:** Validation (equal). **Guowang Li:** Validation (equal). **Haiwei Xu:** Investigation (equal). **Bangxin Liu:** Software (equal). **Bing Yuan:** Software (equal). **Yingjie Zhou:** Writing – review and editing (equal). **Yongjin Li:** Investigation (equal); Writing‐review and editing (equal).

## FUNDING INFORMATION

This study was supported by grants from Special Project on Traditional Chinese Medicine Research in Henan Province (No: 2019ZY1032) and 2023 Luoyang Social Development Public Welfare Special Project (2302020A).

## CONFLICT OF INTEREST STATEMENT

These authors have no conflict of interest to declare.

## Data Availability

The data used to support the findings of this study are included within the article.
